# Effects of Treating Old Rats with an Aqueous *Agaricus blazei* Extract on Oxidative and Functional Parameters of the Brain Tissue and Brain Mitochondria

**DOI:** 10.1155/2014/563179

**Published:** 2014-04-28

**Authors:** Anacharis B. de Sá-Nakanishi, Andréia A. Soares, Andrea Luiza de Oliveira, Jurandir Fernando Comar, Rosane M. Peralta, Adelar Bracht

**Affiliations:** Department of Biochemistry, University of Maringá, Avenida Colombo 5790, 87020900 Maringá, PR, Brazil

## Abstract

Dysfunction of the mitochondrial respiratory chain and increased oxidative stress is a striking phenomenon in the brain of aged individuals. For this reason there has been a constant search for drugs and natural products able to prevent or at least to mitigate these problems. In the present study the effects of an aqueous extract of *Agaricus blazei*, a medicinal mushroom, on the oxidative state and on the functionality of mitochondria from the brain of old rats (21 months) were conducted. The extract was administered intragastrically during 21 days at doses of 200 mg/kg. The administration of the *A. blazei* extract was protective to the brain of old rats against oxidative stress by decreasing the lipid peroxidation levels and the reactive oxygen species content and by increasing the nonenzymic and enzymic antioxidant capacities. Administration of the *A. blazei* extract also increased the activity of several mitochondrial respiratory enzymes and, depending on the substrate, the mitochondrial coupled respiration.

## 1. Introduction

Aging is characterized by a general decline of many physiological functions, with pronounced influence on the cerebral activities [1]. An important theory that explains the diminution of the cerebral activities during aging states that the increased formation of reactive oxygen species (ROS) is an important generator of cellular lesions and disturbances related to old age [2, 3]. The deleterious effects of ROS on biomolecules such as proteins, nucleic acids, and lipid membranes [4] slowly accumulate along the years and have been regarded as an important endogenous factor contributing to aging as well as to the degenerative disturbances associated with the old age [5, 6].

There are numerous routes that lead to the production of ROS, but the mitochondrial energy metabolism is generally recognized as the most important one in most eukaryotic cells [7]. By being the direct intracellular source of ROS the mitochondria are also subject to the direct attack by these molecules [8]. It has been claimed that damage induced by oxidant molecules, including mutations in the mitochondrial DNA, may result in a progressive loss of the cellular capacity of ATP production, cellular degeneration, and eventually cell death [2]. This loss of physiological performance during aging seems to be one of the most important factors involved in the pathogenesis of many disturbances that appear during the old age [9].

The consumption of foods rich in components possessing antioxidant activity has been regarded as a promising measure for the prevention of age related diseases. Mushrooms have been especially recommended due to the fact that they represent a great source of new therapeutic agents [10, 11]. In this sense, the mushroom* Agaricus blazei* Murril, popularly known as* sun mushroom,* has been amply utilized in the form of a medicinal extract for cancer prevention and for treating a variety of conditions such as diabetes, atherosclerosis, hypercholesterolemia, and cardiac diseases [12, 13]. A number of clinical studies have already been conducted, several of them confirming the beneficial effects of* A. blazei*, especially its immunomodulatory activity [14]. Several studies have also shown that* A. blazei* is an important source of potential antioxidant compounds [15, 16], especially phenolics such as gallic acid, syringic acid, and pyrogallol and also polysaccharides [17, 18]. Furthermore,* A. blazei* also contains significant amounts of nucleosides and nucleotides, adenosine, for example, [19], which are important paracrine agents [20, 21] also able to exert neuroprotective actions [22, 23].

Considering, thus, the increased oxidative stress during aging [24–26] and the antioxidant and putative medicinal properties of* A. blazei*, we decided to investigate in detail the influence of an extract of this mushroom on several parameters in the brain tissue of old rats. Besides characterizing the oxidative status of the brain tissue of these rats, cerebral mitochondria were isolated and investigated for their oxidative state and functional properties. The study should answer the question if an aqueous extract of* A. blazei* is or is not able to influence in a positive way the cerebral oxidative state and the mitochondrial functions during aging.

## 2. Materials and Methods

### 2.1. Preparation of the* Agaricus blazei* Aqueous Extract

Basidiomata of* Agaricus blazei* were obtained from a local producer in Maringá, PR, Brazil, in spring 2009. The previously grounded dehydrated basidiomata were submitted to an aqueous extraction as described previously [16] with some minor modifications. The dried basidiomata were milled until obtaining a fine powder. The samples (10 g) were extracted by stirring with 100 mL of water (28°C) at 130 rpm for 3 hours and filtered through Whatman no. 1 paper. The extraction was repeated three times. The filtrates (yield 50%) were lyophilized and stored in freezer until use.

### 2.2. Animals and Treatment

Male* Wistar* rats kept in laboratory cages received water* ad libitum* and a standard chow diet (Nuvilab). The rats were maintained in automatically timed light and dark cycles of 12 hours. Experiments were done with young adult rats (3 months old, weighing 250 to 300 g) and old rats (21 months old, weighing 450 to 500 g). The* A. blazei* extract was administered intragastrically to a group of old rats at a daily dose of 200 mg/kg during 21 days. These rats are labeled as “*A. blazei*-treated old rats” in the graphs and tables. All control rats received saline (0.9% NaCl) during the same period of 21 days. All experiments were done in accordance with the internationally accepted recommendations in the care and use of animals.

### 2.3. Preparation of the Brain Homogenate

Rats were starved for 18 hours and then anesthetized by intraperitoneal injection of thiopental (50 mg/kg). The criterion of anesthesia was the lack of body or limb movement in response to a standardized tail clamping stimulus. The brain of each rat was surgically removed with scissors, clamped with liquid nitrogen, and stored at temperatures under −150°C. The tissue suspension (10% w/v in 0.1 M phosphate buffer, pH 7.4) was homogenized by means of a van Potter-Elvehjem homogenizer. Protein contents were determined with the Folin phenol reagent [27] using bovine serum albumin as standard.

### 2.4. Isolation of Brain Mitochondria

After 18-hour fast the rats were decapitated and their brains removed immediately and cut into pieces with scissors. The fragments were suspended (10% w/v) in a medium containing 0.2 M mannitol, 76 mM sucrose, 10 mM TRIS (pH 7.4), 1 mM ethylene glycol-bis(2-aminoethylether)-*N*,*N*,*N*′,*N*′-tetraacetic acid (EGTA), and 50 mg% fatty acid-free bovine serum albumin. Homogenization of the tissue was done in the same medium using a van Potter-Elvehjem homogenizer. After homogenization the mitochondria were isolated by fractional centrifugation [28, 29]. The isolated mitochondria were suspended in the isolation medium and kept at 0 a 4°C. Protein content was determined with the Folin phenol reagent [27].

### 2.5. Determination of the Total Antioxidant Capacity (TAC)

The total antioxidant capacity of the brain was determined colorimetrically with 2,2′-azino-bis-(3-ethylbenzothiazoline-6-sulphonic acid) (ABTS; [30]). Aliquots from the supernatant of a 10000 g centrifugation of the brain homogenate were added to 0.4 M acetate buffer (pH 5.8) plus 150 *μ*L of a cationic ABTS solution (30 mM acetate buffer, pH 3.6, containing 10 mM ABTS and 4 mM H_2_O_2_). After 5 minutes of incubation in the dark, the absorbance at 734 nm was determined against water. The compound 6-hydroxy-2,5,7,8-tetramethyl-chloraman-2-carboxylic acid (TROLOX) was used as a standard and the results were expressed as *μ*mol TROLOX equivalents per mg protein.

### 2.6. Determination of Lipid Peroxidation, Reduced Glutathione, and Protein Reduced Thiol Contents

The levels of lipid peroxidation were measured in the brain homogenate and in brain mitochondria by means of the TBARS method (thiobarbituric reactive substances). The concentration of lipoperoxides was determined spectrophotometrically at 532 nm using an extinction coefficient (*ε*_532 nm_) of 1.56 × 10^5^ M^−1^ cm^−1^. The results were expressed as nmol malondialdehyde (MDA) per mg protein [31]. The reduced glutathione (GSH) levels of mitochondria and brain homogenate were determined spectrofluorimetrically with o-phthalaldehyde (excitation: 350 nm; emission: 420 nm) [32]. Standards were run in parallel and the glutathione concentration was expressed as nmol per mg protein. The reduced protein thiol groups in the brain homogenate were determined using the compound 5,5′-dithiobis 2-nitrobenzoic acid (DTNB) [33]. The concentration of reduced thiols was calculated using a molar extinction coefficient (*ε*_412 nm_) of 1.36 × 10^4^ M^−1^ cm^−1^ and was expressed as nmol per mg protein.

### 2.7. Reactive Oxygen Species (ROS) Determination

The levels of reactive oxygen species were estimated in aliquots from the supernatants of the 10000 g centrifugation of either disrupted mitochondria or brain homogenate using the reaction with 2′,7′-dichlorofluorescein diacetate (DCFH-DA; [34]). The formation of oxidized 2′,7′-dichloro-fluorescein (DCF) was measured fluorimetrically with excitation at 504 nm and emission at 529 nm. The ROS content was calculated using a standard curve with oxidized dichlorofluorescein (DCF) and the results were expressed as nmol per mg protein. All steps were processed in the dark and a blank containing DCFH-DA was used to correct for autofluorescence.

### 2.8. Determination of Antioxidant Enzymes

The activity of catalase (CAT) was evaluated by measuring spectrophotometrically the decomposition of H_2_O_2_ at 240 nm [35]. Aliquots from the supernatants of the 10000 g centrifugation of either disrupted mitochondria or brain homogenate were added to 1 mL of solution containing 50 mM TRIS (pH 8.0), 0.25 mM EDTA, and 30 mM H_2_O_2_. The drop in absorbance during the first minute of incubation was measured at 25°C. A standard H_2_O_2_ curve was used to calculate the enzyme activity, which was expressed as *μ*mol min^−1^ mg protein^−1^.

The activity of the superoxide dismutase (SOD) was assayed by its capacity to inhibit the auto-oxidation of pyrogallol in alkaline medium, which was monitored spectrophotometrically at 420 nm [36]. One unit of SOD is defined as the amount of enzyme promoting 50% inhibition of pyrogallol auto-oxidation. Aliquots from the supernatants of the 10000 g centrifugation of either disrupted mitochondria or brain homogenate were added to 1 mL of a solution containing 0.2 M TRIS (pH 8.2) and 2 mM EDTA. The reaction was started by adding 0.1 mM pyrogallol. The change in absorbance was monitored, the initial rate computed, and the activity expressed as SOD units per mg protein.

The activity of glutathione peroxidase was determined as the decrease in absorbance at 340 nm due to NADPH oxidation dependent on H_2_O_2_ at 25°C [37]. Aliquots from the supernatants of the 10000 g centrifugation of either disrupted mitochondria or brain homogenate were added to 1.5 mL of a solution containing 40 mM phosphate buffer (pH 7.0), 0.5 mM EDTA, 1.0 mM sodium azide, 1.0 mM reduced glutathione, 1.5 mM NADPH, and 2 units of glutathione reductase. The reaction was initiated by the addition of H_2_O_2_ (0.2 mM) and monitored during 90 seconds. The initial rates were obtained by extrapolation to zero time and the activity computed as nmol min^−1^ mg protein^−1^ using the molar extinction coefficient of NADPH (*ε*_340 nm_) (6.22 × 10^3^ M^−1^ cm^−1^).

The activity of glutathione reductase (GR) was determined as the decrease in absorbance at 340 nm due to the NADPH oxidation [38]. Aliquots from the supernatants of the 10000 g centrifugation of either disrupted mitochondria or brain homogenate were added to 1 mL of a solution containing 50 mM phosphate buffer (pH 8.0), 2 mM EDTA, 0.15 mM NADPH, and 0.5 mM oxidized glutathione (GSSG) at 25°C. The initial rates were obtained by extrapolation to zero time and the activity computed as nmol min^−1^ mg protein^−1^ using the molar extinction coefficient of NADPH (6.22 × 10^3^ M^−1^ cm^−1^).

The activity of glucose 6-phosphate dehydrogenase was measured as the reduction rate of NADP^+^ in the presence of glucose 6-phosphate [38]. Aliquots from the supernatants of the 10000 g centrifugation of brain homogenate were added to 1.5 mL of a solution containing 0.1 M triethanolamine buffer (pH 7.6), 7 mM MgCl_2_, and 1 mM NADP^+^. The reaction at 25°C was initiated by the addition of glucose 6-phosphate (1.0 mM). The increase in absorbance due to NADPH production was monitored during three minutes. The initial rates were obtained by extrapolation to zero time and the activity computed as nmol min^−1^ mg protein^−1^ using the molar extinction coefficient of NADPH (6.22 × 10^3^ M^−1^ cm^−1^).

### 2.9. Determination of Mitochondrial Membrane-Bound Enzymatic Activities

The NADH oxidase and succinate oxidase activities as well as the oxidation of ascorbate mediated by TMPD (*N*,*N*,*N*′,*N*′-tetramethyl-*p-*phenylenediamine) were measured polarographically using freeze-thawing disrupted mitochondria in a medium containing 20 mM TRIS (pH 7.4) [39]. The reactions were initiated by the addition of the corresponding substrates, namely, NADH (1 mM), succinate (10 mM), or ascorbate plus TMPD (10 and 1 mM).

The activity of cytochrome c oxidase was determined spectrophotometrically using freeze-thawing disrupted mitochondria [40]. The rate of ferrocytochrome c oxidation was monitored at 550 nm. The results were expressed as nmol min^−1^ mg protein^−1^ using a molar extinction coefficient (*ε*_550 nm_) of 1.9 × 10^4^ M^−1^ cm^−1^.

The determination of the NADH dehydrogenase in disrupted mitochondria was done spectrophotometrically at 420 nm using ferricyanide as the electron acceptor. The reaction medium contained 20 mM TRIS (pH 7.4), 1.8 *μ*M antimycin, 1 mM NADH, 0.5-0.6 mg mitochondrial protein, and 0.1 mM potassium ferricyanide. The results were expressed as nmol min^−1^ mg protein^−1^ using an extinction coefficient (*ε*_420 nm_) of 1.04 × 10^3^ M^−1^ cm^−1^ [41].

The mitochondrial ATPase activity was quantified by measuring phosphate release from ATP. The reaction medium was 0.2 M sucrose, 10 mM TRIS (pH 7.4), 50 mM KCl 50, 0.2 mM EGTA, and, when appropriate, 100 *μ*M 2,4-dinitrophenol. The reaction was initiated by the addition of 5 mM ATP and interrupted after 20 min incubation at 37°C by the addition of 5% trichloroacetic acid and maintained at 4°C until determination of free phosphate [42].

### 2.10. Determination of Mitochondrial Dehydrogenases

The *α*-ketoglutarate dehydrogenase activity was measured in a reaction medium containing 100 mM phosphate buffer (pH 7.4), 0.2 mM thiamine pyrophosphate, 2 mM NAD^+^, 1 mM MgCl_2_, 0.3 mM dithiothreitol, 0.1% Triton X-100 (v/v), 10 mM *α*-ketoglutarate, and aliquots of freeze-thawing disrupted mitochondria suspensions [43]. The reaction was initiated by the addition of coenzyme A (0.2 mM) and monitored spectrophotometrically as the reduction of NAD^+^ at 340 nm (*ε* = 6.22 × 10^3^ M^−1^ cm^−1^). The initial rate was expressed as nmol min^−1^ mg protein^−1^.

For the determination of pyruvate dehydrogenase, the inactive form (phosphorylated) of the multienzyme complex was converted into the active form (dephosphorylated) by incubating freeze-thawing disrupted mitochondria in a medium containing 20 mM TRIS (pH 7.8), 130 mM KCl, 5 mM potassium phosphate, and 10 mM MgCl_2_. After 5 minutes an aliquot of 50 *μ*L was transferred to the assay medium (1 mL) containing 50 mM TRIS (pH 7.8), 1 mg/mL bovine serum albumin, 50 mM NAD^+^, 2.5 mM coenzyme A, 50 mM MgCl_2_, 5 mM sodium oxalate, 5 mM thiamine pyrophosphate, 100 *μ*M rotenone, 30 mM dithiothreitol 6.5 *μ*M phenazine methosulfate, and 0.6 mM iodonitrotetrazolium chloride. The reaction was started by adding 100 mM pyruvate and the reduction of iodonitrotetrazolium was measured as the increase in absorbance at 500 nm [44]. Activity of the enzyme was expressed as nmol min^−1^ mg protein^−1^ using the molar extinction coefficient of reduced iodonitrotetrazolium (1.24 × 10^4^ M^−1^ cm^−1^ at pH 7.8).

The activity of succinate dehydrogenase was measured in a reaction medium (1 mL) containing 100 mM triethanolamine (pH 8.3), 0.5 mM EDTA, 2 mM KCN, 6.5 *μ*M phenazine methosulfate, 0.6 mM iodonitrotetrazolium, and aliquots from freeze-thawing disrupted mitochondria [45]. The reaction was started by the addition of succinate (10 mM), monitored as the increase in absorbance at 500 nm and expressed as nmol min^−1^ mg protein^−1^ using the molar extinction coefficient of reduced iodonitrotetrazolium (1.93 × 10^4^ M^−1^ cm^−1^ at pH 8.3).

The activity of malate dehydrogenase was measured in a reaction medium (1.5 mL) containing 120 mM phosphate buffer (pH 7.8), 0.25 mM NADH, and aliquots from the supernatant of the 10000 g centrifugation of disrupted mitochondria. The reaction was initiated by the addition of oxaloacetate (0.1 mM) and monitored as the diminution of absorbance at 340 nm [46]. Activity was expressed as nmol min^−1^ mg protein^−1^ using the extinction coefficient of NADH (6.22 × 10^3^ M^−1^ cm^−1^).

The NADP^+^-dependent isocitrate dehydrogenase activity was measured in a medium (1 mL) containing 0.1 M TRIS (pH 7.4), 2 mM MgCl_2_, 2 mM NADP^+^, and aliquots from the supernatant of the 10000 g centrifugation of disrupted mitochondria [47]. The reaction was initiated by the addition of isocitrate (1.25 mM) and monitored as the increase in absorbance at 340 nm. Activity was expressed as nmol min^−1^ mg protein^−1^ using the extinction coefficient of NADPH (6.22 × 10^3^ M^−1^ cm^−1^).

The L-glutamate dehydrogenase activity was measured in a medium (1 mL) containing 50 mM triethanolamine (pH 8.0), 0.1 M ammonium sulfate, 95 *μ*M NADH, 2.5 mM EDTA, 1 mM ADP, and aliquots from the supernatant of the 10000 g centrifugation of disrupted mitochondria [48]. The reaction was initiated by the addition of *α*-ketoglutarate (8.0 mM) and monitored as the increase in absorbance at 340 nm. Activity was expressed as nmol min^−1^ mg protein^−1^ using the extinction coefficient of NADH (6.22 × 10^3^ M^−1^ cm^−1^).

### 2.11. Mitochondrial Oxygen Consumption and Oxidative Phosphorylation

Mitochondrial oxygen consumption was measured polarographically using a teflon-shielded platinum electrode [28, 41]. Mitochondria (*≈*1 mg protein/mL) were incubated in the closed oxygen chamber in a medium (2 mL) containing 0.25 M mannitol, 5 mM sodium phosphate, 10 mM KCl, 0.2 mM EGTA, 50 mg% fatty acid-free bovine serum albumin, 10 mM TRIS-HCl (pH 7.4), and substrates. The latter were succinate (10 mM), *α*-ketoglutarate (10 mM), or pyruvate + L-malate (10 + 1 mM). ADP (0.125 mM) was added at appropriate times. The rates of oxygen consumption, the respiratory control ratio (RC), and the ADP/O ratios were computed from the slopes of the recorder tracings [49]. Rates were expressed as nmol O_2_ min^−1^ mg protein^−1^.

### 2.12. Mitochondrial Membrane Energization

The mitochondrial membrane energization (transmembrane potential) was estimated fluorimetrically using safranin as a fluorescent probe [49, 50]. Mitochondria (1 mg protein) were incubated in a medium (2 mL) containing 0.25 M mannitol, 5 mM potassium phosphate, 10 mM TRIS (pH 7.4), 0.2 mM EGTA, 50 mg% fatty acid-free bovine serum albumin, and 10 *μ*M safranin. Energization was achieved by the introduction of either 50 *μ*M succinate + 2 *μ*M rotenone or 1 mM ATP. Carbonyl cyanide-4-(trifluoromethoxy)phenylhydrazone (FCCP; 10 *μ*M) was added to attain full deenergization. The wavelengths for excitation and emission were 520 and 580 nm, respectively.

### 2.13. Statistics

All results are presented as mean ± mean standard errors. Evaluation of the statistical significance was done by means of the variance analysis (ANOVA) followed by post hoc Student-Newman-Keuls testing. The 5% level (*P* < 0.05) was adopted as the significance criterion.

## 3. Results

### 3.1. Oxidative State of the Brain Tissue and Mitochondria

**Figure 1:**
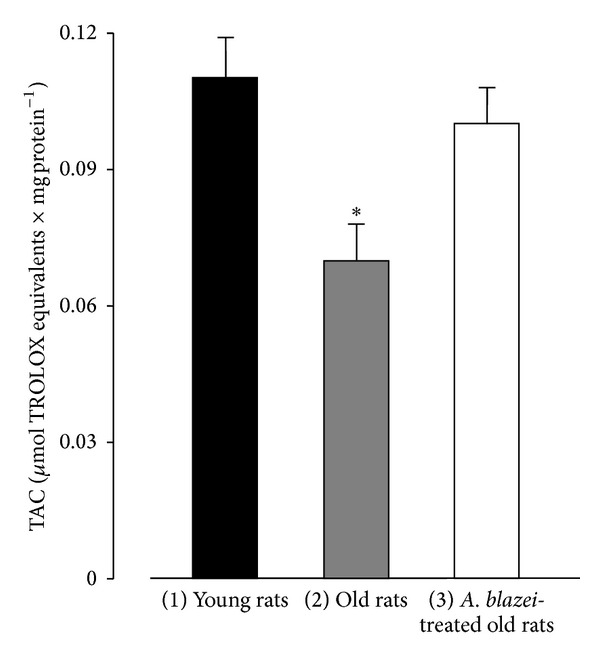
Total antioxidant capacity of the brain homogenates from young, old, and* A. blazei*-treated old rats. The brain homogenate was prepared as described in Section 2. The ABTS assay was used to evaluate the antioxidant capacity of the brain homogenate. The bars represent the mean ± mean standard errors of 5 (young), 8 (old), and 5 (*A. blazei*-treated) rats. The asterisk (∗) indicates *P* ≤ 0.05 for the comparison between (1) and (2) and (2) and (3), according to ANOVA followed by Student-Newman-Keuls post hoc testing.

The total antioxidant capacities of samples from the supernatant of the 10000 g centrifugation of the brain homogenate from young (3 months old) and old rats (21 months) without treatment and treated with the* A. blazei* extract are shown in Figure 1. The ABTS assay revealed a substantial decline of the total antioxidant capacity, the difference between young and old rats amounting to −36%. Figure 1 also shows that the 21-day treatment with the* A. blazei* extract was able to restore the antioxidant capacity of the old rats' brains to a level very close to that of young rats.

**Figure 2:**
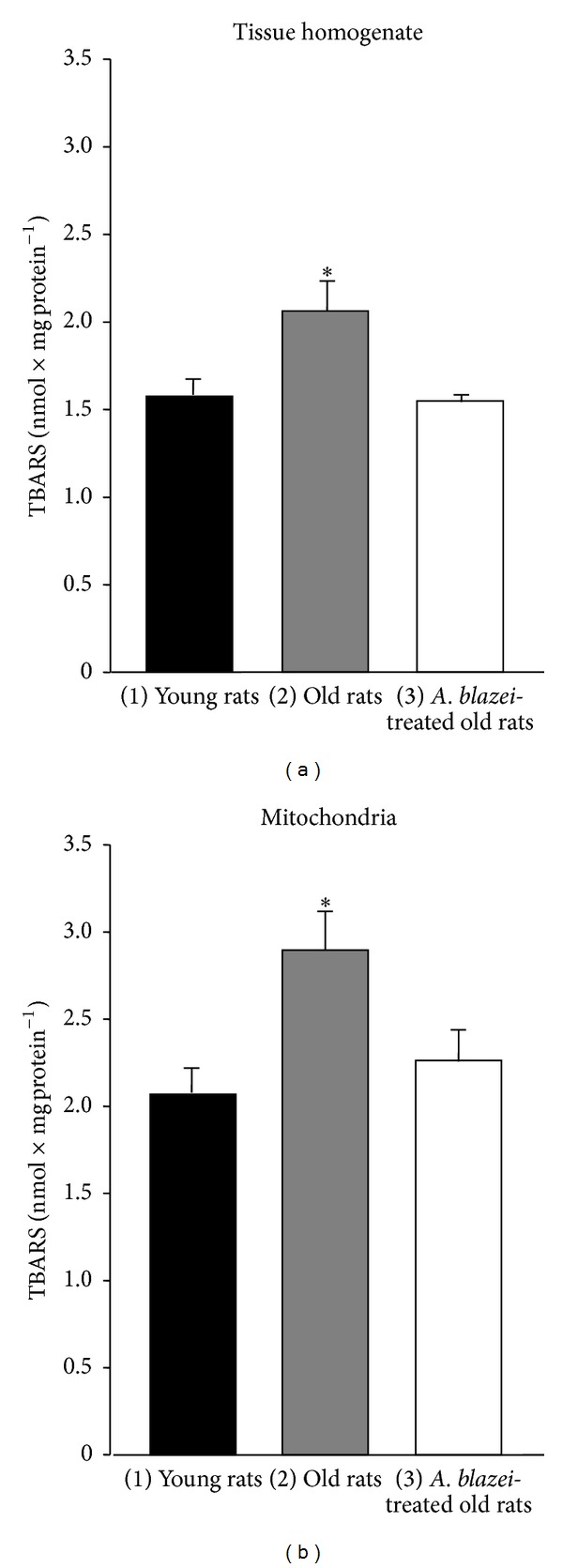
Lipid peroxidation levels of brain homogenate and brain mitochondria from young, old, and* A. blazei*-treated old rats. The brain homogenate and the mitochondria were prepared as described in Section 2. The lipid peroxidation levels were evaluated as the thiobarbituric acid reactive substances (TBARS). The bars represent the mean ± mean standard errors of 5 (young), 5 (old), and 4 (*A. blazei*-treated) rats for the homogenate and of 6 (young), 6 (old), and 7 (*A. blazei*-treated) rats for the mitochondria. The asterisks (∗) indicate *P* ≤ 0.05 for the comparison between (1) and (2) and (2) and (3), according to ANOVA followed by Student-Newman-Keuls post hoc testing.

The lipid peroxidation levels were measured in both the total brain homogenate and the mitochondrial fraction. The results are shown in Figure 2. Lipid peroxidation was clearly higher in the brain homogenate of old rats (+33%) when compared to that of young rats (Figure 2(a)). A more pronounced difference (+40%) was found in the mitochondrial fraction (Figure 2(b)). The* A. blazei* extract treatment, however, restored almost completely the lipid peroxidation level of the total brain tissue of old rats to that found in young rats. A similar effect of the extract treatment was found for the mitochondrial fraction, though restoration was not complete in this case.

**Figure 3:**
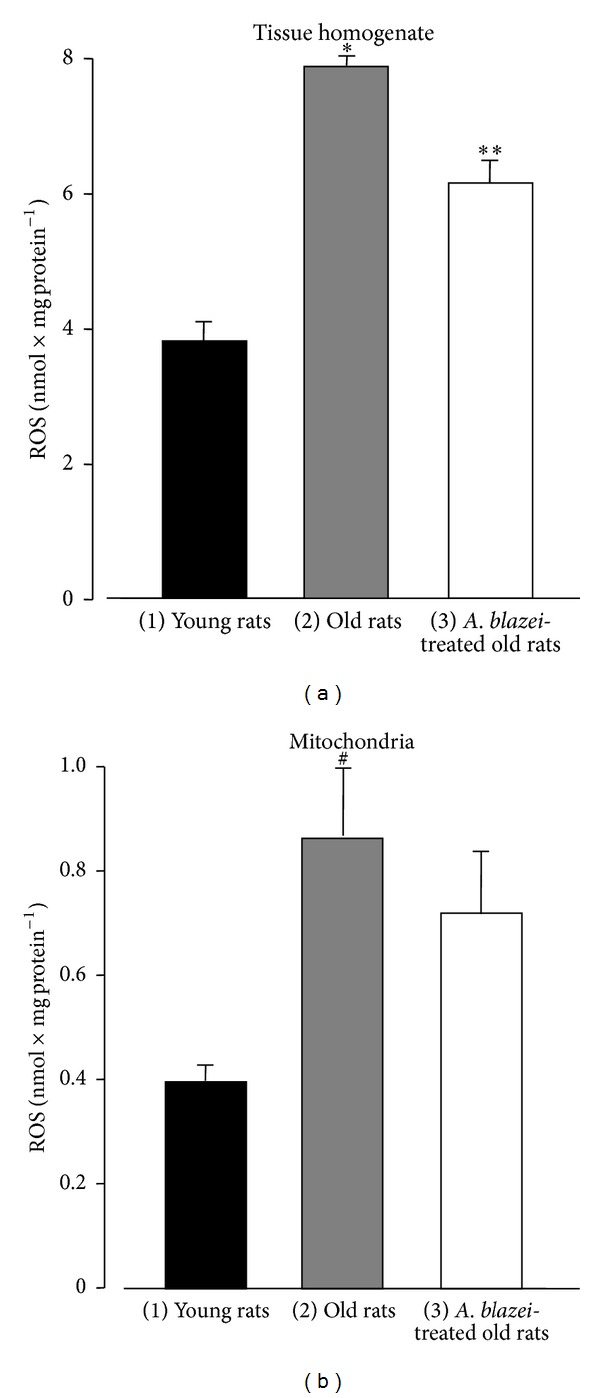
Reactive oxygen species (ROS) levels of brain homogenate and brain mitochondria from young, old, and* A. blazei*-treated old rats. The brain homogenate and the mitochondria were prepared as described in Section 2. The ROS levels were evaluated by means of the dichlorofluorescein diacetate method. The bars represent the mean ± mean standard errors of 5 (young), 7 (old), and 6 (*A. blazei*-treated) rats for the homogenate and of 4 (young), 6 (old), and 5 (*A. blazei*-treated) rats for the mitochondria. *P ≤ 0.05 for the comparison between (1) and (2) and (2) and (3), according to ANOVA followed by Student-Newman-Keuls post hoc testing; **P ≤ 0.05 for the comparison between (3) and (1); ^#^ *P* ≤ 0.05 for the comparison between (2) and (1).

The levels of reactive oxygen species in both total homogenate and mitochondria can be seen in Figure 3. Old rats presented an enormous increase relative to young rats, +106% in the total homogenate (Figure 3(a)) and +119% in the mitochondria (Figure 3(b)). Treatment of old rats with the* A. blazei* extract was partially successful. For the total brain tissue it caused a diminution of 22%. For the mitochondria a diminishing tendency was also found, but lacking statistical significance at the 5% level.

**Figure 4:**
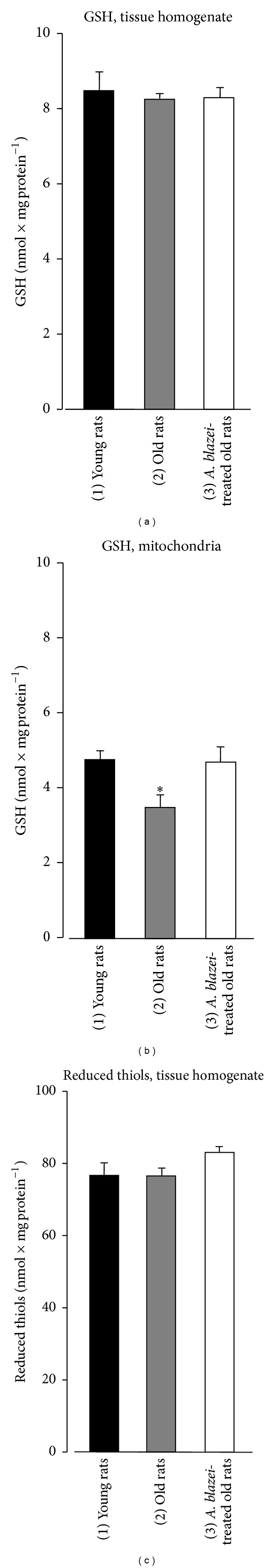
Reduced glutathione (GSH) levels and protein thiol groups of brain homogenate and reduced glutathione (GSH) levels of brain mitochondria from young, old, and* A. blazei*-treated old rats. The brain homogenate and the mitochondria were prepared as described in Section 2. The GSH levels were evaluated spectrofluorimetrically by o-phthalaldehyde method. The reduced thiol groups were measured with 5,5′-dithiobis 2-nitrobenzoic acid (DTNB) method. The bars represent the mean ± mean standard errors; in panel (a) (tissue homogenate) the *n* values were 5 (young), 9 (old), and 6 (*A. blazei*-treated); in panel (b) the *n* values were 6 (young), 6 (old), and 6 (*A. blazei*-treated) and in panel (c) *n* values were 5 (young), 6 (old), and 5 (*A. blazei*-treated). *P ≤ 0.05 for the comparison between (1) and (2) and (2) and (3), according to ANOVA followed by Student-Newman-Keuls post hoc testing.

Figure 4 summarizes the results obtained when the GSH levels were measured as well as the protein reduced thiol levels. Aging did not affect the GSH concentration of the total cerebral tissue nor had the* A. blazei* treatment any influence (Figure 4(a)). In the mitochondria, however, aging reduced the GSH levels by 26% (Figure 4(b)). The* A. blazei* treatment restored the mitochondrial GSH levels of old rats to the levels found in young rats. The reduced thiol levels of the total homogenate (Figure 4(c)) were not affected by aging and* A. blazei* treatment.

### 3.2. Antioxidant Enzymes of the Brain Tissue and Mitochondria

The activities of five antioxidant enzymes were measured and the results are summarized in Table 1. The catalase activity in the homogenate (supernatant of 10,000 g centrifugation) was not affected by aging, but the* A. blazei* extract treatment induced an increase of 38% in old rats. Singularly, in mitochondria of old rats the catalase activity was considerably smaller than that in young rats (−47%), a condition that was completely abolished by the* A. blazei* treatment.

Superoxide dismutase in the brain homogenate supernatant showed some tendency toward diminution in old when compared to young rats. Treatment with the* A. blazei* extract, on the other hand, produced an increase in the superoxide dismutase activity of old rats (+20%) which was statistically significant. The changes in the superoxide dismutase activity were more pronounced in the mitochondria, where a clear decrease was found upon aging (−24%) and a recovery in consequence of the* A. blazei* extract treatment.

The glutathione peroxidase activities in the homogenate supernatant and mitochondria behaved quite differently upon aging: the activity of this enzyme increased in the total brain homogenate (+49%) but remained the same in the mitochondria. A further increase (+29%) was found in the homogenate upon the* A. blazei* extract treatment, whereas a nonsignificant increasing tendency was apparent in the mitochondria. The combined increases in the brain glutathione peroxidase activity caused by aging and* A. blazei* treatment, thus, amounted to 92%.

Aging was without significant effect on the tissue activity of glutathione reductase, but the glucose 6-phosphate dehydrogenase was substantially diminished in old rats (−26%). The* A. blazei* extract treatment produced a partial recovery.

### 3.3. Mitochondrial Enzyme Activities

The mitochondrial enzyme activities corresponding to individual proteins or to segments of the respiratory chain that were measured in the present work are listed in Table 2. With respect to their behavior during aging and their response to the* A. blazei* extract treatment they may be divided into three groups. The first group comprises those enzymes that were clearly diminished upon aging: NADH dehydrogenase (−16%), succinate dehydrogenase (−45%), pyruvate dehydrogenase (−27%), and *α*-ketoglutarate dehydrogenase (−36%). Excepting pyruvate dehydrogenase, the* A. blazei* extract treatment resulted in enzyme levels that were equal or even superior to those found in young rats. For pyruvate dehydrogenase this tendency was much less pronounced. The second group of enzymes in Table 2 comprises only cytochrome c oxidase, whose activity was not affected by aging, but which suffered a very pronounced increase in consequence of the* A. blazei* extract treatment (+90%). And the third group comprises the enzymes that were altered neither by aging nor by the* A. blazei* extract treatment. These enzymes were malate dehydrogenase, glutamate dehydrogenase, and isocitrate dehydrogenase (NADP^+^-dependent).

Table 3 shows the results of experiments in which three oxidases were measured polarographically using freeze-thawing disrupted brain mitochondria. Aging diminished NADH oxidase by 21%, succinate oxidase by 17%, and the TMPD mediated ascorbate oxidation “TMPD-ascorbate oxidase” by 30%. Treatment of the old rats with the* A. blazei* extract had a positive effect only on the TMPD mediated ascorbate oxidation (+24%).

The ATPase activity measurements are summarized in Table 4. ATP hydrolysis was measured in three different systems. Aging caused diminutions of 33%, 22%, and 36%, respectively, in the ATPase activities of coupled mitochondria, uncoupled mitochondria, and freeze-thawing disrupted mitochondria. A significant positive effect of the* A. blazei* treatment was found only for the ATPase activity of freeze-thawing disrupted mitochondria (+24%).

### 3.4. Mitochondrial Respiration and Membrane Energization

The respiratory activity of intact brain mitochondria was measured using three different substrates, namely, succinate, pyruvate + L-malate, and *α*-ketoglutarate. The classical protocol was used in which the mitochondria were initially incubated in the closed oxygraph chamber without exogenous substrates. After stabilization of the very small respiratory rate, substrates were added and the mitochondria were allowed to respire under these conditions for 2-3 minutes. This respiration is labeled as the −ADP rate of oxygen uptake in Table 5. This phase was followed by the addition of a limited amount of ADP. The ADP stimulated respiration is labeled as the +ADP rate of oxygen uptake in Table 5. Table 5 reveals that the −ADP respiration was not significantly affected by aging when the substrates were succinate and pyruvate + L-malate. A diminution of 39% occurred, however, when *α*-ketoglutarate was the substrate. Coupled respiration (+ADP respiration), on the other hand, was significantly reduced by aging when succinate and pyruvate + L-malate were the substrates, the diminution amounting to 20 and 24%, respectively. The diminution caused by aging in the *α*-ketoglutarate coupled respiration lacked statistical significance. Treatment of old rats with the* A. blazei* extract was effective only in restoring the ADP-dependent respiration when succinate was the substrate. With pyruvate + L-malate as substrates a tendency toward restoration was apparent, but it lacked statistical significance. The ADP/O ratios were not significantly diminished by aging, nor were the respiratory control ratios affected. With respect to these parameters the only significant action of the* A. blazei* treatment was an increase in the ADP/O ratio when *α*-ketoglutarate was the substrate.

**Figure 5:**
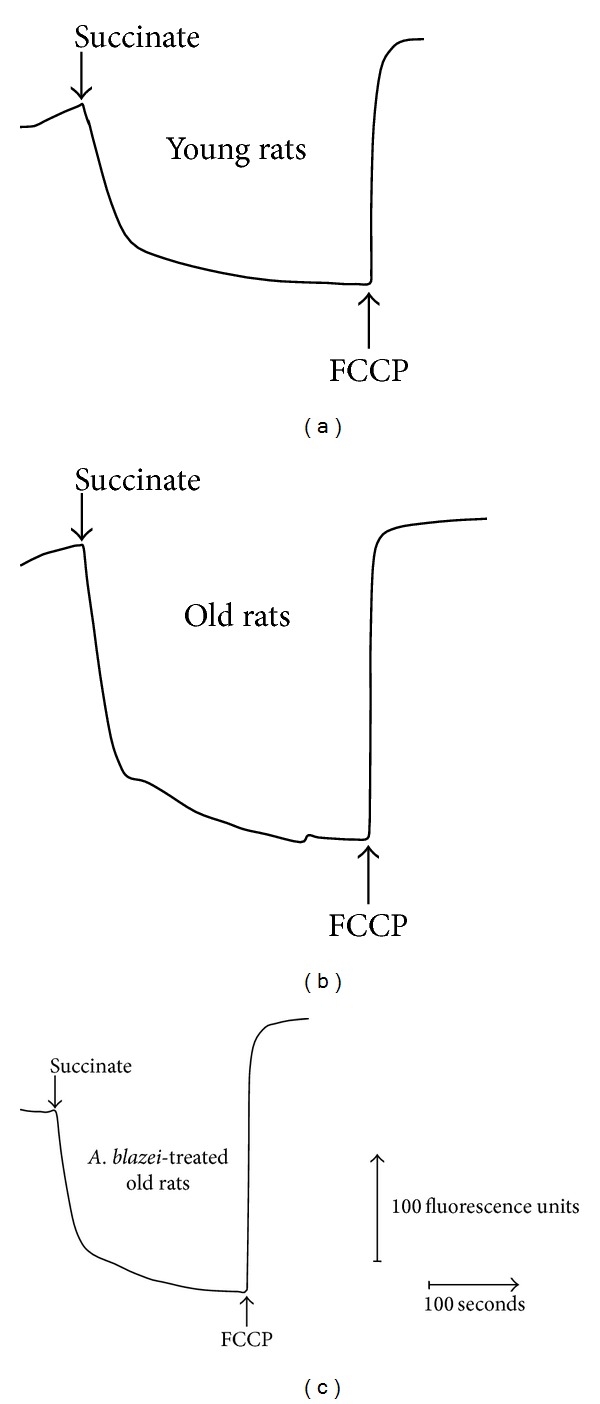
Changes in safranin fluorescence due to succinate in incubations containing brain mitochondria. Mitochondria (1 mg protein) were incubated in a medium containing (2 mL) containing 0.25 M mannitol, 5 mM potassium phosphate, 10 mM TRIS (pH 7.4), 0.2 mM EGTA, 50 mg% fatty acid-free bovine serum albumin, and 10 *μ*M safranin. Succinate and FCCP were added as indicated. The wavelengths for excitation and emission were 520 and 580 nm, respectively.

The membrane energization of brain mitochondria was assayed using safranin as a fluorescent probe [49, 50]. Typical traces are shown in Figure 5 and the mean results of the evaluations are listed in Table 6. The numbers in Table 6 represent the changes in fluorescence that occurred upon the addition of the uncoupler carbonyl cyanide-4-(trifluoromethoxy)phenylhydrazone (FCCP) which was done after stabilization of the fluorescence intensity due to succinate or ATP addition (Figure 5). With mitochondria of old rats the addition of succinate caused a more intense decrease in safranin fluorescence (24%) when compared to young rats. This effect was further enhanced by the* A. blazei* extract treatment (22% over nontreated old rats). Energization due to ATP addition, on the other hand, tended to be smaller in mitochondria of old rats when compared to the mitochondria of young rats, but the experimental variation was quite pronounced so that statistical significance could not be demonstrated. The* A. blazei* treatment of old rats, however, had also an increasing effect on the ATP-induced membrane energization.

## 4. Discussion

Diminution of mitochondrial enzyme activities and respiration upon aging is a phenomenon that has been observed in several studies including rat brain, liver, and cardiac mitochondria [25, 26, 51–55]. There are also studies with human liver and skeletal muscle mitochondria [56, 57]. In the majority of these studies, however, either enzyme activities or the respiratory activity with various substrates were measured. An exception is a study with human liver mitochondria [56], in which coupled respiration and some oxidases were measured. The simultaneous measurement of these parameters, however, is important for establishing possible rate-limiting relationships. In the present study with brain mitochondria several enzyme activities have been measured in addition to the respiratory activity of intact mitochondria, so that our data offer the opportunity of discussing the way by which these variables are related. Among the enzymes measured in the present work succinate dehydrogenase was the most strongly affected by aging as it was 45% diminished in old compared to young rats. This reduction, however, did not reflect in a similar diminution in the respiratory rates of intact mitochondria. In fact, no diminution was found in the absence of exogenously added ADP, a situation in which the phosphorylation rate limits respiration. When this limitation was abolished by the addition of ADP a 20% diminution of respiration was observed. This diminution was similar to that found in disrupted mitochondria (succinate oxidase), namely, 17%. The diminution of 20% would also correspond to the inhibition of oxidative phosphorylation in mitochondria of old rats, a relatively modest figure if one takes into account both the diminutions of the succinate dehydrogenase activity (−45%) and the ATPase/ATP-synthase activity (−33%), which are presumed to contribute simultaneously to the rate of coupled respiration. In the case of the substrate *α*-ketoglutarate the discrepancy between enzyme activity and coupled respiration is even more pronounced. Old rats revealed a 36% diminished *α*-ketoglutarate activity. Coupled respiration with *α*-ketoglutarate, however, was only minimally affected in mitochondria of old rats to the point that the difference was not even statistically significant. A more close correlation between enzyme inhibition and ADP-dependent respiration was found when pyruvate + L-malate were used as substrates. The pyruvate dehydrogenase was 27% smaller in mitochondria of old rats and the coupled respiration was 24% smaller. Malate dehydrogenase, as a near-equilibrium enzyme (very high activity), is not expected to exert any rate control activity. Clearly, taking the observations as a whole, no prediction about the actual impairment of coupled respiration can be made based on the diminution of enzyme activities because it is difficult to infer a priori the rate-limiting step for a given phenomenon. This occurs probably because there are many factors influencing the actual rate of ADP phosphorylation in intact mitochondria, including the membrane energization which, by the way, was increased in mitochondria of old rats at least when succinate was the substrate. If higher membrane energization, as detected by the safranin probe, represents increased ionic concentration gradients, this could also be meaning an additional drive for oxidative phosphorylation, partly compensating enzyme deficiencies.

The* A. blazei* treatment was quite successful in improving the oxidative state of the brain of old rats. Our observations are consistent with similar effects of the same* A. blazei* extract on the injury caused by paracetamol in both hepatic and brain tissue [18]. The* A. blazei* extract was particularly able to bring back the levels of lipid peroxidation (TBARS) of old rats to those found in young rats. This action can, in principle at least, be attributed to the free-radical scavenging ability of several constituents of* A. blazei*, as, for example, the phenolics [58]. The three phenolics that have been identified in* A. blazei*, gallic acid, syringic acid, and pyrogallol, have also demonstrated to possess pronounced antioxidant activities [17]. They are probably present in the aqueous extract used in the present study if one takes into account their pronounced hydrophilic character.

Important constituents of* A. blazei* that are infrequently mentioned in the specialized literature are adenosine and other nucleosides and nucleotides which are quite abundant in this mushroom [19]. Nucleosides and nucleotides are purinergic agents and purinergic effects of an* A. blazei* extract have been recently demonstrated to occur in the rat liver [19]. Adenosine, but possibly also other activators of A_1_ purinergic receptors, confers cytoprotection in the cardiovascular and central nervous systems by activating cell surface adenosine receptors [22, 59]. Activation of these receptors, in turn, is postulated to activate antioxidant enzymes via protein kinase C phosphorylation of the enzymes or of intermediates that promote activation [22]. It is thus possible that the increased activities of antioxidant enzymes caused the* A. blazei* treatment in old rats were partly caused by its contents in adenosine or other purinergic agents.

Antioxidant action can also be expected from the polysaccharides that are present in* A. blazei* [60, 61]. In this respect it must be mentioned that the hepatoprotective action of partially purified fungal polysaccharides (including antioxidant effects) has been recently demonstrated [62, 63]. Further active components of* A. blazei* might also include oligopeptides. In fact an oligopeptide from* A. blazei*, rich in Pro, Lys, and Phe and possessing antioxidant activity, has been recently described [64].

The* A. blazei* treatment was also successful in increasing the activities of several mitochondrial enzymes in old rats especially the succinate dehydrogenase, *α*-ketoglutarate dehydrogenase, NADH dehydrogenase, and the cytochrome c oxidase. The latter was nearly doubled by the* A. blazei* treatment. These results are close to those obtained in a study in which old rats were treated with* Ganoderma lucidum* extracts using an experimental schedule similar to that of the present work [55]. Furthermore, the* A. blazei *treatment enhanced both the succinate- and ATP-dependent membrane energization of the mitochondrial membrane. In spite of all these effects the influence of the treatment on the respiratory rates of intact mitochondria in the presence and absence of ADP was relatively modest. Only the succinate-driven respiration in the presence of exogenous ADP was significantly increased in such a way as to approach the respiration rates found in brain mitochondria of young rats. No doubt that this event, which is mainly the consequence of the succinate dehydrogenase stimulation by the* A. blazei* treatment, represents a gain in terms of the rat brain energetics. On the other hand, with respect to the other dehydrogenases, the question must be raised about the possible physiological consequences of their stimulations by the* A. blazei* extract treatment. At least for the *α*-ketoglutarate and pyruvate dehydrogenases their increases may be related to the improvement of the oxidative state of the mitochondria as evidenced by the increased mitochondrial GSH levels found in treated old rats. Enhanced activities of the *α*-ketoglutarate and pyruvate dehydrogenases are expected to increase the NADH/NAD^+^ ratio which, in turn, also leads to an increased NADPH nucleotide transhydrogenase activity with a concomitant increase in the NADPH/NADP^+^ ratio. The consequence will be an increased regeneration of GSH via glutathione reductase and also a more efficient removal of H_2_O_2_ via glutathione peroxidase [65, 66].

The mechanisms by which the* A. blazei* extract increases the activities of several enzymes linked to energy metabolism in the mitochondria of old rats cannot be inferred with certainty from the data obtained in the present work. However, it is likely that the antioxidant properties of the extract played an important role. This proposition bases on the fact that many alterations in enzymatic activities in the brain of old rats are accompanied by oxidative modifications in proteins such as carbonylations and nitrations [67]. In principle such modifications can be prevented by improving the antioxidant defenses of the cells, a task for which the* A. blazei* extract is well equipped as already discussed above. It is also possible that components of the extract promote the expression of specific proteins. Important candidates for these actions are the purinergic agents of the extract, adenosine and others [19]. Purinergic signalling is very important in the neuronal tissue [21] and it is said that the levels of adenosine increase when there is an imbalance between the rates of energy use and the rates of energy delivery [21].

In conclusion, the experiments of the present work confirm the widespread notion that aging increases oxidative stress in the brain [2, 4, 24, 34] and that it also negatively affects several mitochondrial enzyme activities involved in energy metabolism [24, 51], though not always a precise correlation between coupled respiration and enzyme activities was apparent. And, more important, the results show that treatment with an aqueous extract of* Agaricus blazei* can improve the oxidative state of the brain tissue and can also reverse some of the deleterious effects of the aging process on mitochondrial oxidative enzymes. It remains to be demonstrated if these effects also occur in humans during aging. If confirmed, an extract of this mushroom could be incorporated into the diet as a supplement able to stimulate the body defense against oxidative stress.

## Figures and Tables

**Table 1:** Effects of the *A. blazei* extract treatment on the activity of the antioxidant enzymes in the brain tissue and in brain mitochondria of old rats. All enzymes were evaluated spectrophotometrically as described in Materials and Methods. The values represent the mean ± standard errors of the mean.

Parameters	Experimental groups		
	(1) Young rats	(2) Old rats	(3) *A. blazei*-treated old rats
Brain homogenate (supernatant of 10,000 g centrifugation)			
Catalase (*μ*mol min^−1^ mg protein^−1^)	12.90 ± 0.57 (*n* = 5)	12.89 ± 0.74 (*n* = 7)	17.78 ± 0.86** (*n* = 5)
Superoxide dismutase (units mg protein^−1^)	1.86 ± 0.12 (*n* = 5)	1.70 ± 0.07^#^ (*n* = 8)	2.04 ± 0.07 (*n* = 6)
Glutathione peroxidase (nmol min^−1^ mg protein^−1^)	20.42 ± 0.60 (*n* = 5)	30.47 ± 3.09* (*n* = 6)	39.29 ± 2.46^##^ (*n* = 5)
Glutathione reductase (nmol min^−1^ mg protein^−1^)	16.09 ± 0.67 (*n* = 4)	14.51 ± 1.05 (*n* = 8)	14.93 ± 0.76 (*n* = 6)
Glucose 6-phosphate dehydrogenase (nmol min^−1^ mg protein^−1^)	34.23 ± 1.63 (*n* = 5)	25.43 ± 0.82* (*n* = 8)	29.29 ± 0.79^##^ (*n* = 6)

Brain mitochondria			
Catalase (*μ*mol min^−1^ mg protein^−1^)	5.72 ± 0.52 (*n* = 4)	3.05 ± 0.12* (*n* = 5)	6.25 ± 1.08 (*n* = 5)
Superoxide dismutase (units mg protein^−1^)	2.14 ± 0.11 (*n* = 5)	1.63 ± 0.10* (*n* = 5)	2.10 ± 0.12 (*n* = 5)
Glutathione peroxidase (nmol min^−1^ mg protein^−1^)	20.51 ± 1.48 (*n* = 5)	20.52 ± 1.32 (*n* = 5)	24.98 ± 2.32 (*n* = 5)

*P < 0.05 for (2) versus (1) and (2) versus (3) (ANOVA and Student-Newman-Keuls post hoc testing).

**P < 0.05 for (3) versus (2) and (3) versus (1) (ANOVA and Student-Newman-Keuls post hoc testing).

^#^ *P* < 0.05 for (2) versus (3).

^##^ *P* < 0.05 for (3) versus (1).

**Table 2:** Effects of the *A. blazei* extract treatment on several enzymatic activities of brain mitochondrial from old rats. All enzymes were evaluated spectrophotometrically as described in Materials and Methods. The values represent the mean ± standard errors of the mean.

Enzymes	Experimental groups		
	(1) Young rats	(2) Old rats	(3)* A. blazei-*treated old rats
nmol min^−1^ mg protein^−1^			
NADH dehydrogenase	698.90 ± 29.99 (*n* = 3)	588.17 ± 16.51* (*n* = 5)	752.12 ± 36.35 (*n* = 6)
Succinate dehydrogenase	14.22 ± 1.84 (*n* = 6)	7.83 ± 0.94* (*n* = 5)	14.57 ± 0.95 (*n* = 6)
*α*-Ketoglutarate dehydrogenase	17.24 ± 1.48 (*n* = 6)	10.98 ± 0.54* (*n* = 5)	16.13 ± 0.98 (*n* = 6)
Pyruvate dehydrogenase	23.75 ± 2.37 (*n* = 4)	17.26 ± 0.27^#^ (*n* = 5)	21.03 ± 1.22 (*n* = 6)
Cytochrome c oxidase	287.20 ± 31.30 (*n* = 5)	264.53 ± 16.23 (*n* = 5)	501.32 ± 25.09** (*n* = 4)
Malate dehydrogenase	7540 ± 560 (*n* = 6)	6780 ± 770 (*n* = 5)	6980 ± 270 (*n* = 6)
Isocitrate dehydrogenase	26.68 ± 1.79 (*n* = 6)	25.80 ± 2.89 (*n* = 5)	27.57 ± 1.31 (*n* = 6)
Glutamate dehydrogenase	183.6 ± 11.9 (*n* = 6)	179.5 ± 19.9 (*n* = 5)	179.9 ± 8.4 (*n* = 6)

*P < 0.05 for (2) versus (1) and for (2) versus (3) (ANOVA and Student-Newman-Keuls post hoc testing).

^#^ *P* < 0.05 for (2) versus (1) (ANOVA and Student-Newman-Keuls post hoc testing).

**P < 0.05 for (3) versus (2) and (3) versus (1) (ANOVA and Student-Newman-Keuls post hoc testing).

**Table 3:** Effects of the *A. blazei* extract treatment on the oxidation of NADH, succinate, and TMPD-ascorbate by disrupted brain mitochondria of old rats. The NADH oxidase, succinate oxidase, and oxidation of ascorbate mediated by TMPD were measured polarographically using freeze-thawing disrupted mitochondria as described in Materials and Methods. The values represent the mean ± standard errors of the mean.

Enzymatic activities	Experimental groups		
	(1) Young rats	(2) Old rats	(3) *A. blazei-*treated old rats
nmol O_2_ min^−1^ mg protein^−1^			
NADH oxidase	58.56 ± 2.19 (*n* = 6)	46.12 ± 4.84^#^ (*n* = 6)	46.44 ± 4.77** (*n* = 7)
Succinate oxidase	38.12 ± 1.47 (*n* = 6)	31.50 ± 1.53^#^ (*n* = 5)	32.27 ± 1.32 (*n* = 5)
TMPD-ascorbate oxidation	113.89 ± 5.75 (*n* = 4)	79.40 ± 3.03^#^ (*n* = 5)	98.66 ± 6.25 (*n* = 5)

^#^ *P* < 0.05 for (2) versus (1) (ANOVA and Student-Newman-Keuls post hoc testing).

**P < 0.05 for (3) versus (1) ANOVA and Student-Newman-Keuls post hoc testing).

**Table 4:** Effects of the *A. blazei* extract treatment on the ATPase activity of mitochondria from old rats. The ATPase activity was quantified by measured phosphate release from ATP as described in Materials and Methods. The values represent the mean ± standard errors of the mean.

Conditions	Experimental groups		
	(1) Young rats	(2) Old rats	(3)* A. blazei-*treated old rats
nmol P_*i*_ released min^−1^ mg protein^−1^			
Intact mitochondria	85.04 ± 2.11 (*n* = 5)	56.77 ± 2.13^#^ (*n* = 5)	62.29 ± 3.15** (*n* = 6)
Intact mitochondria + 2,4-dinitrophenol	97.28 ± 4.16 (*n* = 5)	75.44 ± 6.55^#^ (*n* = 5)	78.89 ± 4.84** (*n* = 6)
Freeze-thawing disrupted mitochondria	90.49 ± 2.97 (*n* = 4)	58.11 ± 2.85* (*n* = 4)	71.86 ± 4.42** (*n* = 5)

*P < 0.05 for (2) versus (1) and for (2) versus (3) (ANOVA and Student-Newman-Keuls post hoc testing).

^#^ *P* < 0.05 for (2) versus (1) (ANOVA and Student-Newman-Keuls post hoc testing).

**P < 0.05 for (3) versus (1) (ANOVA and Student-Newman-Keuls post hoc testing).

**Table 5:** Effects of the *A. blazei* extract treatment on the respiration of brain mitochondria of old rats. The oxygen consumption, ADP/O ratio, and respiration coefficient (RC) were assessed in the mitochondria as described in Materials and Methods. The values represent the mean ± standard errors of the mean.

Substrate	Experimental groups	Rate of oxygen uptake (nmol min^−1^ mg protein^−1^)		ADP/O	RC
		−ADP	+ADP		
Succinate (10 mM)	(1) Young rats	16.11 ± 1.27 (*n* = 6)	51.65 ± 2.06 (*n* = 6)	1.54 ± 0.02 (*n* = 6)	2.67 ± 0.14 (*n* = 6)
	(2) Old rats	14.16 ± 1.477 (*n* = 6)	41.6 ± 2.68* (*n* = 6)	1.22 ± 0.14 (*n* = 5)	2.30 ± 0.32 (*n* = 6)
	(3) *A. blazei*-treated old rats	16.01 ± 0.976 (*n* = 7)	52.20 ± 3.50 (*n* = 6)	1.24 ± 0.16 (*n* = 6)	2.54 ± 0.27 (*n* = 6)

Pyruvate + L-malate (10 mM + 1 mM)	(1) Young rats	9.95 ± 0.26 (*n* = 6)	40.47 ± 2.44 (*n* = 6)	1.78 ± 0.10 (*n* = 6)	1.92 ± 0.10 (*n* = 6)
	(2) Old rats	9.64 ± 0.71 (*n* = 5)	30.71 ± 3.03^#^ (*n* = 5)	1.66 ± 0.28 (*n* = 5)	1.85 ± 0.16 (*n* = 5)
	(3) *A. blazei*-treated old rats	10.64 ± 0.54 (*n* = 7)	35.48 ± 2.12 (*n* = 7)	1.67 ± 0.04 (*n* = 6)	1.95 ± 0.18 (*n* = 7)

*α*-Keto-glutarate (10 mM)	(1) Young rats	4.85 ± 0.46 (*n* = 6)	18.50 ± 1.39 (*n* = 6)	1.88 ± 0.08 (*n* = 6)	2.63 ± 0.39 (*n* = 6)
	(2) Old rats	2.99 ± 0.62^#^ (*n* = 3)	15.53 ± 1.53 (*n* = 3)	2.17 ± 0.21 (*n* = 3)	3.22 ± 0.90 (*n* = 3)
	(3) *A. blazei*-treated old rats	3.51 ± 0.15 (*n* = 3)	16.39 ± 0.81 (*n* = 3)	2.60 ± 0.12** (*n* = 3)	4.43 ± 2.29 (*n* = 3)

*P < 0.05 for (2) versus (1) and (2) versus (3) (ANOVA and Student-Newman-Keuls post hoc testing).

^#^ *P* < 0.05 for (2) versus (1) (ANOVA and Student-Newman-Keuls post hoc testing).

**P < 0.05 for (3) versus (1) (ANOVA and Student-Newman-Keuls post hoc testing).

**Table 6:** Effects of the *A. blazei* extract treatment on the mitochondrial membrane energization by forward (succinate) and reverse (ATP) electron flow. The mitochondrial membrane energization (transmembrane potential) was estimated fluorimetrically using safranin as a fluorescent probe. Intact mitochondria (1 mg protein) were incubated in a medium as described in Materials and Methods. The values represent the mean ± standard errors of the mean.

Energizing agent	Experimental groups		
	(1) Young rats	(2) Old rats	(3) *A. blazei*-treated old rats
	Fluorescence units		
Succinate	150.7 ± 6.5 (*n* = 4)	187.5 ± 11.7* (n = 5)	228.2 ± 8.07^#^ (n = 6)
ATP	81.3 ± 17.4 (*n* = 7)	48.3 ± 3.9 (n = 4)	130.5 ± 20.0** (n = 6)

*P < 0.05 for (2) versus (1) and (2) versus (3) (ANOVA and Student-Newman-Keuls post hoc testing).

^#^ *P* < 0.05 for (3) versus (1) (ANOVA and Student-Newman-Keuls post hoc testing).

**P < 0.05 for (3) versus (2) (ANOVA and Student-Newman-Keuls post hoc testing).
